# A genetic model of CEDNIK syndrome in zebrafish highlights the role of the SNARE protein Snap29 in neuromotor and epidermal development

**DOI:** 10.1038/s41598-018-37780-4

**Published:** 2019-02-04

**Authors:** Valeria Mastrodonato, Galina Beznoussenko, Alexandre Mironov, Laura Ferrari, Gianluca Deflorian, Thomas Vaccari

**Affiliations:** 1 IFOM, The FIRC Institute of Molecular Oncology, via Adamello 16, 20139 Milan, Italy; 2 IEO, European Institute of Oncology, via Adamello 16, 20139 Milan, Italy; 3 University of Milan, Department of Biosciences, Via Celoria 26, 20133 Milan, Italy

## Abstract

Homozygous mutations in *SNAP29*, encoding a SNARE protein mainly involved in membrane fusion, cause CEDNIK (Cerebral Dysgenesis, Neuropathy, Ichthyosis and Keratoderma), a rare congenital neurocutaneous syndrome associated with short life expectancy, whose pathogenesis is unclear. Here, we report the analysis of the first genetic model of CEDNIK in zebrafish. Strikingly, homozygous *snap29* mutant larvae display CEDNIK-like features, such as microcephaly and skin defects. Consistent with Snap29 role in membrane fusion during autophagy, we observe accumulation of the autophagy markers p62 and LC3, and formation of aberrant multilamellar organelles and mitochondria. Importantly, we find high levels of apoptotic cell death during early development that might play a yet uncharacterized role in CEDNIK pathogenesis. Mutant larvae also display mouth opening problems, feeding impairment and swimming difficulties. These alterations correlate with defective trigeminal nerve formation and excess axonal branching. Since the paralog Snap25 is known to promote axonal branching, Snap29 might act in opposition with, or modulate Snap25 activity during neurodevelopment. Our vertebrate genetic model of CEDNIK extends the description *in vivo* of the multisystem defects due to loss of Snap29 and could provide the base to test compounds that might ameliorate traits of the disease.

## Introduction

SNAP29 (Synaptosomal-associated protein 29) is a member of the conserved SNARE (Soluble NSF, N-ethylmaleimide-sensitive factor, Attachment Protein REceptor) family^1^, which regulates membrane fusion during intracellular trafficking processes^2^. Snap29 possesses an acidic NPF motif at its N-terminus followed by two SNARE domains required for fusion in association with a target-SNARE protein, such as a Syntaxin, and a vesicle-associated SNARE protein, or Vamp. Several recent studies in human cells and in *Drosophila melanogaster* revealed a key requirement of Snap29 in the regulation of macroautophagy (autophagy here after)^3–6^. Autophagy is a degradative pathway involved in the disposal of damaged organelles, long-lived proteins or toxic aggregates^7^. During autophagy Snap29, Syntaxin17 and VAMP8, mediate the fusion between mature autophagosomes and lysosomes^4–6^. Beyond autophagy, Snap29 is involved in a number of membrane fusion events within the cell, taking part in diverse trafficking processes, such as endocytosis, recycling and specialized forms of secretion, some of which require the NPF motif^8^. Finally, Snap29 could contribute to non-trafficking processes such as regulation of cell division. In fact, in *Drosophila* Snap29 is repurposed as a kinetochore component, and in both *Drosophila* and mammalian cells, Snap29 depletion affects chromosome segregation, ultimately leading to formation of micronuclei and to cell death^9^.

Despite the widespread use of Snap29 in several trafficking and non-trafficking processes, complete loss of human Snap29 (SNAP29) does not cause embryonic lethality. Indeed, homozygous inactivating mutations in the human *SNAP29* gene are responsible for *Ce*rebral *D*ysgenesis, *N*europathy, *I*chthyosis, palmoplantar *K*eratoderma (CEDNIK; OMIM # 609528), a rare autosomal recessive syndrome characterized by congenital neurological and dermatological alterations. These include palmoplantar keratoderma and ichthyosis, microcephaly, neurogenic muscle atrophy, reduced peripheral nerve conduction, corpus callosum abnormalities and cortical dysplasia. The severity of the traits determines a radical shortening of lifespan ranging from neonatal lethality to 12 years^10–12^. Owing to the many cellular functions of Snap29, the pathogenesis of CEDNIK is largely unknown.

Skin biopsies of CEDNIK patients revealed a thickened stratum corneum (hyperkeratosis). Further ultrastructural analysis of the skin of CEDNIK mouse models and zebrafish embryos transiently depleted of Snap29, show accumulation of empty lamellar granules in upper epidermal layers^12–14^. In physiologic conditions, granules containing lipid and protein are transported from the Golgi apparatus to the surface of the epidermis, suggesting that trafficking supporting normal skin development and homeostasis might be defective in CEDNIK patients^15^. Consistent with this, CEDNIK patient-derived fibroblasts show a fragmented Golgi and an altered morphology of early and recycling endosomes^11^.

CEDNIK patients also present severe nervous system development defects, including pachygyria, polymicrogyria and psychomotor retardation. The first two manifestations are thought to refer respectively to abnormal neuronal migration in the developing brain and to brain cortex malformations, due to an excessive number of small and fused cortical convolutions^12^. Since CEDNIK patient-derived neural tissue biopsies are not readily available, the nervous system defects were not investigated in CEDNIK patients. Thus, the nature and consequences of loss of SNAP29 in nervous system development remain elusive. However, Snap29 is likely directly involved in synaptic transmission. In fact, Snap29 is found at synapses of rat hippocampal neurons, where it acts as a negative modulator of synaptic vesicle exocytosis. Indeed, Snap29 has been found to compete with α-SNAP for the binding with the SNARE complex formed by Snap25, Syntaxin1A and VAMP2, thus inhibiting SNARE complex disassembly that is required for synaptic vesicles recycling. Consistent with this, overexpression of Snap29 in presynaptic neurons inhibits synaptic transmission, whereas knockdown of Snap29 promotes it^16,17^.

Here, we have established to our knowledge the first genetic model of CEDNIK in zebrafish. With it, we have investigated *in vivo* the cellular, tissue and organismal consequences of lack of Snap29 on vertebrate development. We described multisystemic alterations that could be explained by loss of most of Snap29 described functions. In addition, we report alteration of neuro-muscular development that might shed light on ill-explored aspects of CEDNIK.

## Results

### Establishment of a genetic CEDNIK disease model in zebrafish

**Figure 1:**
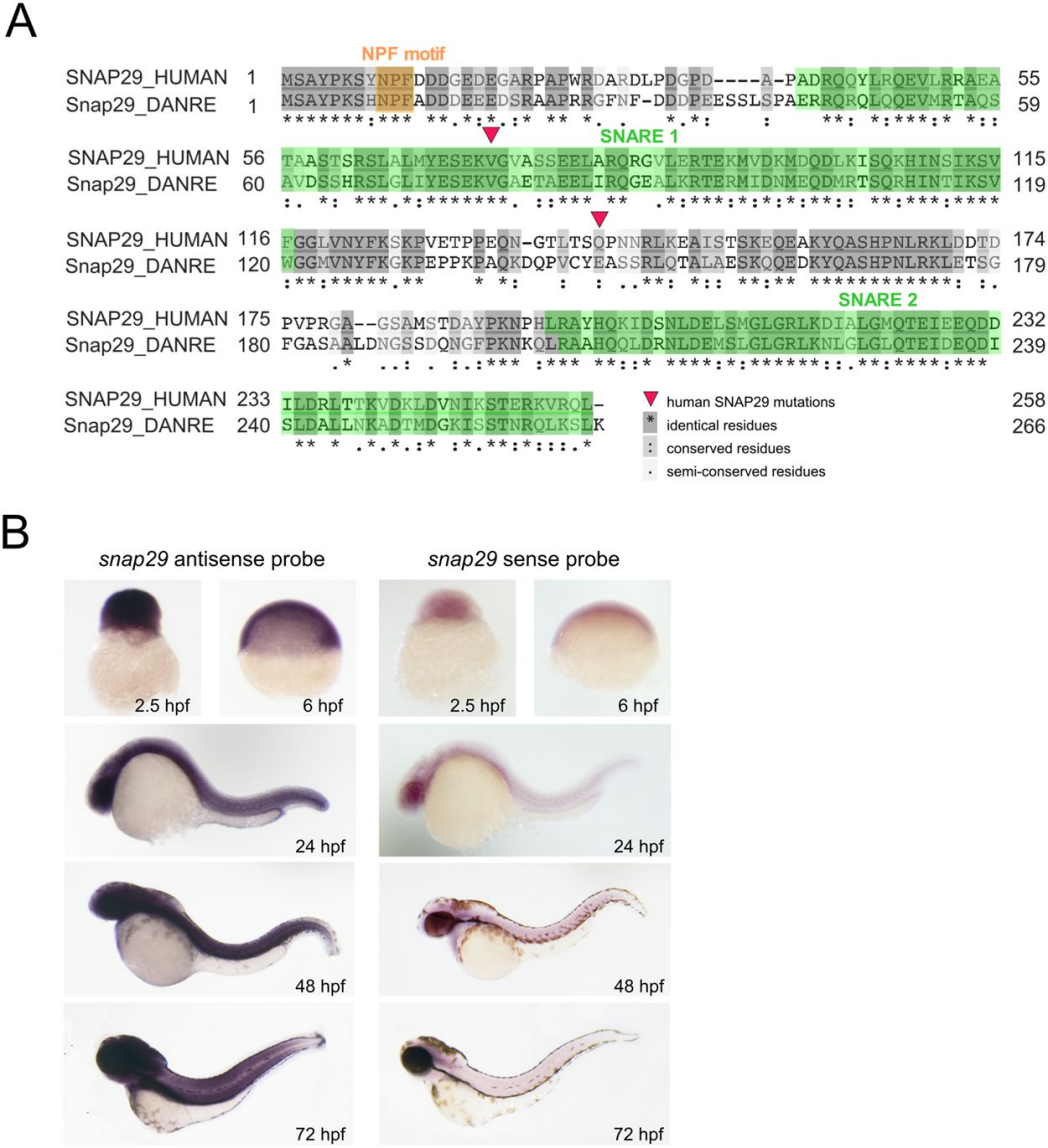
Snap29 conservation and expression in zebrafish. (**A**) Protein sequence alignment of human and zebrafish Snap29 from UniProt (http://www.uniprot.org). Red triangles refer to two SNAP29 mutations described in CEDNIK patients. Amino acid residues are shaded according to their degree of conservation, as described in the legend. (**B**) Whole-mount *in situ* hybridization with *snap29* antisense and sense probes on zebrafish embryos at the indicated developmental stages.

To understand whether zebrafish could represent a suitable model organism for human CEDNIK syndrome, we first analyzed protein sequence conservation of zebrafish Snap29 with its human homolog. Zebrafish Snap29 displays overall 46% identity at the amino acid level with the human counterpart. It also possesses all the domains found in SNAP29, namely an acidic NFP motif at its N-terminus and two SNARE domains (Fig. 1A). The position of reported nonsense mutations associated to CEDNIK (red triangles, Fig. 1A)^10,12^ introduce stop codons that are expected to lead to the production of proteins truncated respectively within the first SNARE domain and before the second SNARE domain (Fig. 1A).

To characterize *snap29* expression during zebrafish embryogenesis, we first performed whole-mount *in situ* hybridization and RT-PCR. Consistent with previous evidence^18^, these experiments indicated that the *snap29* mRNA is ubiquitously expressed from maternal stages (2.5 hours post fertilization, hpf) onwards (Figs 1B; S1A).

To reevaluate previous evidence from depletion of *snap29* in zebrafish, we took advantage of an already published *snap29* splice-blocking Morpholino (MO)^14^. To test the efficiency of *snap29* MO depletion at different developmental stages, we performed reverse transcriptase PCR (RT-PCR) of developing embryos (Fig. S1A). In MO-injected embryos, from 24 hpf onwards, we observed retention of an intron in the *snap29* transcript caused by the splicing block. In agreement with our *in situ* experiment (Fig. 1B), but in contrast with previously published evidence^14^, we observed expression of *snap29* mRNA as early as 2.5 hpf (Fig. S1A). However, morphological analysis of *snap29* morphants at 60 hpf produced phenotypes, such as a lighter pigmentation at the level the head and less regular distribution of melanocytes in the tail compared to uninjected embryos (Fig. S1B), which are similar to those published in Li *et al*.^14^.

**Figure 2:**
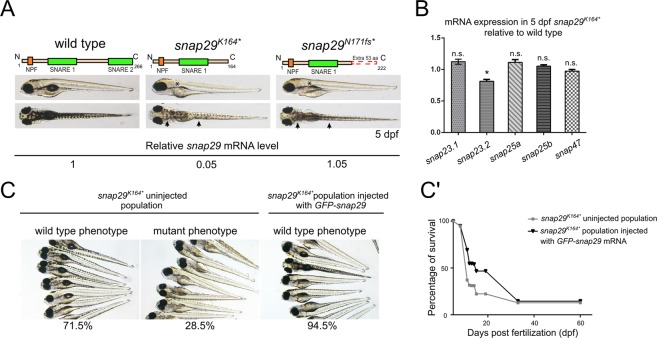
Generation of *snap29* mutants and rescue. (**A**) Lateral and dorsal views of 5 dpf larvae of wild type, ENU *snap29*^K164*^ mutant and CRISPR/Cas9 *snap29*^*N170fs*^ mutants with schematic representation of predicted proteins. Asterisks in lateral views indicate lack of an inflated swim bladder, while arrows in dorsal views point to weaker pigmentation in mutants, compared to wild type. *snap29* mRNA relative expression measured in extracts of 5 dpf wild type, *snap29*^K164*^ and *snap29*^*N170fs*^ larvae, and normalized on *gapdh* is reported below the images. *snap29*^K164*^ mutants show a reduction of *snap29* mRNA of about 95% compared to wild type, while no significant reduction is detectable in *snap29*^*N170fs*^. (**B**) mRNA expression of *snap23.1*, *snap23.2, snap25a, snap25b* and *snap47* measured in extracts of 5 dpf *snap29*^K164*^ relative to wild type. *gapdh* is used as normalizer. The bars in the graph show means and standard errors of the mean (SEM) of three technical replicates. *P*-values were computed by multiple t-test. *P ≤ 0.05. (**C**) Groups of 5 dpf larvae from the progeny of *snap29*^K164*^ heterozygous mating. Uninjected larvae were classified according to the pigmentation phenotype. The *snap29*^K164*^ uninjected population is composed by 71.5% of wild type phenotype larvae and by 28.5% of mutant phenotype larvae. The *snap29*^K164*^ injected population consists of the 94.5% of phenotypically wild type larvae, demonstrating that *GFP-snap29* mRNA injection rescues the mutant phenotype. (**C’**) Survival curve of *snap29*^K164*^ populations uninjected and injected with *GFP-snap29* mRNA. The percentage of survival refers to alive zebrafish larvae or adults recovered over time from the initial number of fertilized embryos. Injected *snap29*^K164*^ mutants show an increase of survival compared to uninjected animals.

Because of the inherent limitation of Morpholino approaches^19^, to attempt to establish a CEDNIK model we requested an uncharacterized *snap29* ENU mutant available from the European Zebrafish International Resource Center (EZRC). Moreover, we generated a CRISPR/Cas9 mutant. Both mutants, called *snap29*^K164*^ and *snap29*^*N171fs*^ respectively, introduce stop codons that lead to the production of truncated Snap29 proteins lacking the SNARE 2 domain, similar to one of the human reported CEDNIK mutations (Fig. 2A). In particular, in *snap29*^K164*^ the stop codon after K164 is the result of a non-sense (T > A) mutation, while the stop codon of *snap29*^*N171fs*^ occurs after an Indel, causing a frameshift (fs) starting after the codon producing N171 (Fig. 2A). Macroscopic analysis of 5 days post fertilization (dpf) larvae revealed that in both crosses of the heterozygous fish, roughly 25% of the progeny showed an uninflated swim bladder (Fig. 2A, asterisks) and, similar to morphants, a lighter pigmentation of the head and of the trunk regions (Fig. 2A, arrowheads). In addition, larvae displaying these phenotypes die at 9 dpf. Genotyping of these animals revealed that they were homozygous respectively for *snap29*^K164*^ and *snap29*^*N171fs*^ (data not shown).

Quantification of *snap29* mRNA level by qPCR in both mutants, selected according to the phenotype described above, showed that *snap29*^*N171fs*^ homozygous larvae express *snap29* mRNA level comparable to control, while *snap29*^K164*^ homozygous larvae display a reduction of its expression of more than 95% (Fig. 2A), suggesting that they are subjected to nonsense-mediated mRNA decay. Since the level of *SNAP29* transcript in CEDNIK patient-derived fibroblasts is strongly reduced compared to control^12^, we decided to select *snap29*^K164*^ homozygous mutants as a valid CEDNIK model. However, since other reported patients have not been analyzed at the mRNA stability level^11^, *snap29*^*N171fs*^ might eventually represent a model for these, or for future patients showing normal expression of *SNAP29* mRNA.

To understand whether *snap29* paralogs might substitute for the observed loss of Snap29 in mutants, we determined their expression by qPCR. We found that, relative to control, levels of expression of *snap29* paralogs are not changed in *snap29*^K164*^ samples (Fig. 2B), suggesting that compensation or redundancy of function are unlikely.

These changes in pigmentation of the skin in mutants and morphants, and in swim bladder appearance in mutants are phenotypes that do not appear to correlate to CEDNIK traits, so we analyzed them first. To this end, we visualized melanocytes of *snap29*^K164*^ mutants. Compared to wild type, we did not appreciate any difference in melanocytes morphology. However, we noticed a reduced amount of melanin accumulated within each melanocyte (Fig. S2). These data suggest that Snap29 might regulate pigment production, possibly at the level of trafficking to melanosomes, which are lysosomal derivatives^20^. A second, prominent feature of *snap29*^K164*^ mutant larvae is the lack of swim bladder inflation (Figs 2C, S3A, upper row). Swim bladder inflation in zebrafish occurs after 5 dpf and is required for free feeding after yolk consumption^21^, since it allows buoyancy and active swimming. To test whether mutant fish are able to feed, we administered Rhodamine Dextran-containing food to 6 dpf wild type and *snap29*^K164*^ mutant larvae. We observed that guts of *snap29*^K164*^ mutant larvae were empty as they are those of wild type animals that were not fed (Fig. S3A, lower row). The inability to feed can be one of the causes determining the precocious lethality observed at 9 dpf in *snap29* mutants. Since the swim bladder is inflated by air gulped from the water surface through the mouth opening^22^, we wondered whether *snap29*^K164*^ mutants present normal buccal cartilages. Alcian Blue staining of ventral cranial cartilages did not highlight defects in their organization and differentiation (Fig. S3B).

To validate our model, we next tested whether the lighter pigmentation, the lack of swim bladder inflation and the lethality observed in homozygous larvae were caused by *snap29* mutation. To determine this, we injected mRNA encoding a zebrafish GFP-tagged *snap29* mRNA, that is efficiently translated into protein (Fig. S4), in a population of one-cell embryos derived from the mating of *snap29*^K164*^ heterozygous fish (Fig. 2C). In the uninjected population, roughly a quarter of 5 dpf larvae displayed the mutant phenotypes, while the remaining larvae were wild type-like. In sheer contrast, the vast majority of the injected population of larvae exhibited a wild type-like phenotype, as expected for Mendelian inheritance (Fig. 2C). Moreover, the injected population displayed increased survival, compared to the uninjected (Fig. 2C’). By genotyping a portion of the population, we found that homozygous larvae were still present in the injected population at 14 dpf, but not at 40 dpf (data not shown). The survival curve analysis suggests that the rescue by transient expression of GFP-Snap29, extends the lifespan of at least a pool of homozygous larvae up to 33 dpf. These data establish *snap29*^K164*^ homozygous larvae as the first validated genetic mutant in zebrafish. We next investigated the defects caused by loss of Snap29 to test whether they correlate with loss of previously characterized Snap29 functions and whether they reproduce CEDNIK syndrome traits.

### *snap29* mutant larvae recapitulate aspects of loss of Snap29 function and of CEDNIK syndrome

Considering recent reports that revealed that Snap29 regulates a late step of autophagy in multiple organisms^4,5,23^, we determined whether autophagy is altered in our *snap29* zebrafish mutant larvae. To this end, we measured the level of the autophagy marker LC3 in protein extracts of 5 dpf larvae by Western blot. We observed a mild increase in both LC3II and the autophagy adapter p62 in *snap29*^K164*^ mutants (Fig. S5A,B). Since LC3II is associated to mature autophagosomes^24^, this result suggests an impairment in autophagy clearance, in line with previous reports^4,5^. In addition, we also found a punctate localization of the p62 in the brain of 4 dpf *snap29*^K164*^ mutants (Fig. S5C), as previously observed in tissues and cells lacking most of Snap29^5,9^. Consistent with the presence of autophagic defects, ultra-structural analysis of the skin of 7 dpf larvae shows that *snap29*^K164*^ mutants accumulate multilamellar organelles (MLOs), which are composed of concentric lipidic membrane layers^25^, within intercellular cavities (Fig. S5D, red asterisk). The formation of MLOs is associated to pathological conditions, such as lysosomal storage diseases^26,27^, and is associated with defective autophagosome clearance^28^ and lysosomal cholesterol accumulation^29^. The occasional presence of MLOs in intercellular cavities suggests that defective autophagy might result in secretion of aberrant organelles, as reported in *Drosophila Snap29* mutants^4^. Together, these data confirm *in vivo* the existence of defects in the process of autophagy in homozygous *snap29*^K164*^ mutant zebrafish.

As one of the most characteristic features of CEDNIK patients is the keratoderma and ichthyosis^12^, two major skin alterations, we investigated possible defects in the epidermis of *snap29*^K164*^ mutant. Unlike mammals, the zebrafish skin is composed of two layers of cells only, the basal layer (Fig. S6A, pseudo-coloring in magenta) resting on the basement membrane (BM; Fig. S6A, pseudo-coloring in orange) and the periderm, which will differentiate keratinocytes (Fig. S6A, pseudo-coloring in yellow)^30^. The ultra-structural analysis of 7 dpf larvae highlighted that *snap29*^K164*^ mutants show a thinner peridermal layer composed of more elongated cells, compared to wild type, while they possess similar basal layers morphology (quantified in Fig. S6A’). In addition, the skin of a wild type sample shows an ordered pattern of structures that will originate adult scales precursors (SP; Fig. S6A, red asterisks), which is lost in the *snap29*^K164*^ mutant (quantified in Fig. S6A”). The reduced size of the peridermal layer in *snap29*^K164*^ mutants was further confirmed by the strongly reduced amount of the peridermal marker Keratin by Western blot analysis (Fig. S6B). Finally, higher magnifications of peridermal cells show looser adherens junctions in the *snap29*^K164*^ mutant compared to wild type (Fig. S6C, white arrows), and increased intercellular space (Fig. S6C, red asterisk). In summary, these results are in contrast with the reported skin hyperkeratosis observed both in CEDNIK patient biopsy^12^ and in conditional CEDNIK mouse mutants^13^. However, the loss of SP organization and the defective junctions are in line with the loss of skin organization integrity observed in patients^13^. Overall, these data suggest that *snap29*^K164*^ zebrafish mutants recapitulate one major CEDNIK trait, as well as the loss of an intensely-studied function of Snap29. Despite this, some differences exist with respect to patients and mammals, in particular at the level of the skin.

### Snap29 is required to determine correct neuromuscular development and function

**Figure 3:**
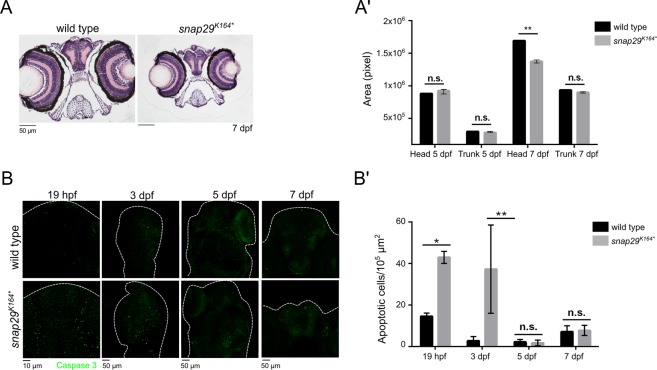
*snap29* mutant as a CEDNIK disease model. (**A**) Hematoxylin-eosin stained paraffin sections of heads of 7 dpf larvae. Note the reduced size of the head in *snap29*^K164*^ mutant animals compared to wild type. (**A’**) Quantification of the area of head and of the trunk sections measured in wild type and *snap29*^K164*^ mutant respectively at 5 dpf and 7 dpf. At 7 dpf, *snap29*^K164*^ mutants show a significant reduction of head area compared to wild type. Bars in the graph show means and standard deviations. *P*-values were obtained by unpaired t-test. **P ≤ 0.01, n = 3. (**B**) Comparable maximum projections of wild type and *snap29*^K164*^ mutant heads at 19 hpf, 3 dpf, 5 dpf and 7 dpf stained with anti-cleaved Caspase 3 to reveal presence of apoptotic cells. White dashed-lines were drawn to highlight head morphology. (**B’**) Quantification of the number of apoptotic cells per 10^5^ μm^2^, measured in wild type and *snap29*^K164*^ mutants respectively at 19 hpf, 3 dpf 5 dpf and 7 dpf. 19 hpf and 3 dpf *snap29*^K164*^ mutants show a significant increase of apoptotic cells compared to wild type. The bars in the graph show means and standard deviations. *P*-values were obtained unpaired t-test with Welch’s correction. *P ≤ 0.05, **P ≤ 0.01, n = 2–9.

It has been reported by clinicians that CEDNIK patients display a severe central nervous system development deficiency and suffer from neurogenic and skeletal muscle hypotonia^31^. However, the pathogenesis of these symptoms has not been investigated. By analyzing hematoxylin-eosin stained paraffin sections of the head of *snap29*^K164*^ mutant larvae at 7 dpf, we observed a prominent microcephaly (Fig. 3A; quantified in 3A’). In contrast, we did not find significant differences in size at 5 dpf (Fig. 3A’). Head area measurements were compared to trunk areas that were not significantly different both at 5 and 7 dpf, suggesting that the effect is not due to a developmental delay. To investigate the origin of the microcephaly, we determined amounts of apoptotic cells during development. Interestingly, we found that the heads of *snap29*^K164*^ mutants at the stage of 20 somites (19 hpf) and 3 dpf exhibit a sharp increase in the expression of the apoptotic marker cleaved Caspase 3, compared to controls, while this is not observed in 5 dpf or 7 dpf mutant animals (Fig. 3B; quantified in 3B’). To exclude that size differences are due to altered rates of proliferation, we determined the level of expression of phospho-Histone H3 (pH3) at 19 hpf and 7 dfp. We found no statistically significant difference (Fig. S7A, quantified in B). These data suggest that microcephaly, a characteristic trait of CEDNIK patients, might be caused at least partially by uncompensated elimination of defective cells during early development.

**Figure 4:**
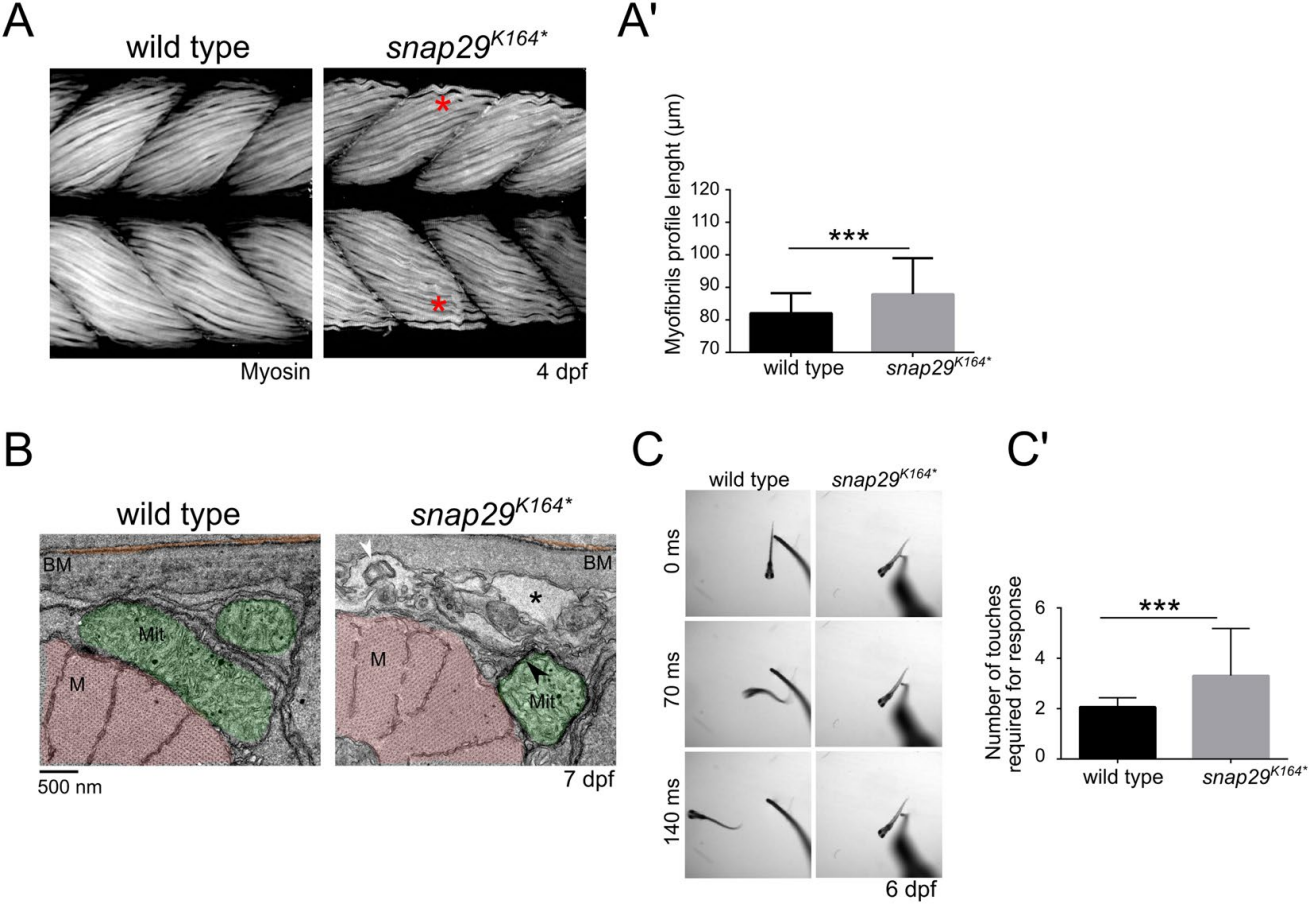
Snap29 supports correct muscle development and function. (**A**) Lateral views of muscles in the trunk of 4 dpf wild type and *snap29*^K164*^ mutant larvae, stained with anti-Myosin heavy chain antibody. Compared to wild type, *snap29*^K164*^ mutants present less compacted and ordered filaments (red asterisk). (**A’**) Quantification of the length of the profile of superficial myofibrils measured in wild type and *snap29*^K164*^ mutant 4 dpf larvae. *snap29*^K164*^ mutants show a significant increase in myofibril length compared to wild type. The bars in the graph show means and standard deviations. *P*-values were derived from Mann-Whitney test. ***P ≤ 0.001, n = 41–45. **(B**) Electron microscopy cross-sections of muscle fibers. *snap29*^K164*^ contain MLO (white arrowhead) within extracellular spaces (asterisks) just beneath the basement membrane and a mitochondria surrounded by a double membrane (black arrowhead), which are not present in wild type animals. M: myofibrils, Mit: mitochondria, BM: basement membrane. (**C**) Selected frames from movies of a wild type and a *snap29*^K164*^ mutant larva at 6 dpf recorded for 1 minute after a touch stimulus on the tail. (**C’**) Quantification of the number of touches required to evoke an escape response. Most of the analyzed *snap29*^K164*^ mutants require two or more touches to respond. The graph reports means and standard deviations. *P*-values were obtained by Mann-Whitney test. ***P ≤ 0.001, n = 6.

Because CEDNIK patients are hypotonic, we then analyzed the pattern of the muscle fibers in the trunk muscles of *snap29*^K164*^ mutant larvae. These show less compacted and ordered filaments compared to wild type (Fig. 4A, red asterisks, quantified in A’). Consistent with muscle fiber disorganization, EM analysis of muscles revealed the presence of intracellular cavities with MLO (Fig. 4B, black asterisk and white arrowhead) within muscle fibers, between myofibrils (M) and the basement membrane (BM) of *snap29*^K164*^ mutants. Furthermore, *snap29*^K164*^ mutants possess mitochondria (Mit) that are included in a double membrane (Fig. 4B, black arrowhead), suggesting a potential alteration of mitophagy. These phenotypes, possibly due to defects in membrane trafficking processes, were recently observed in muscular atrophy patients^32^.

To evaluate the neuromuscular activity of wild type and mutant larvae, we performed a touch-evoked escape response assay. By stimulating tails of 6 dpf mutants with a pipette tip, we observed on average swim-away response of wild type larvae 70 milliseconds (ms) after a single touch, while *snap29*^K164*^ mutants responded very poorly (Fig. 4C, Movies S1 and 2). Quantification of touch stimuli required to trigger escape, revealed that most of *snap29*^K164*^ mutant larvae require two or more touches to swim away (Fig. 4C’).

**Figure 5:**
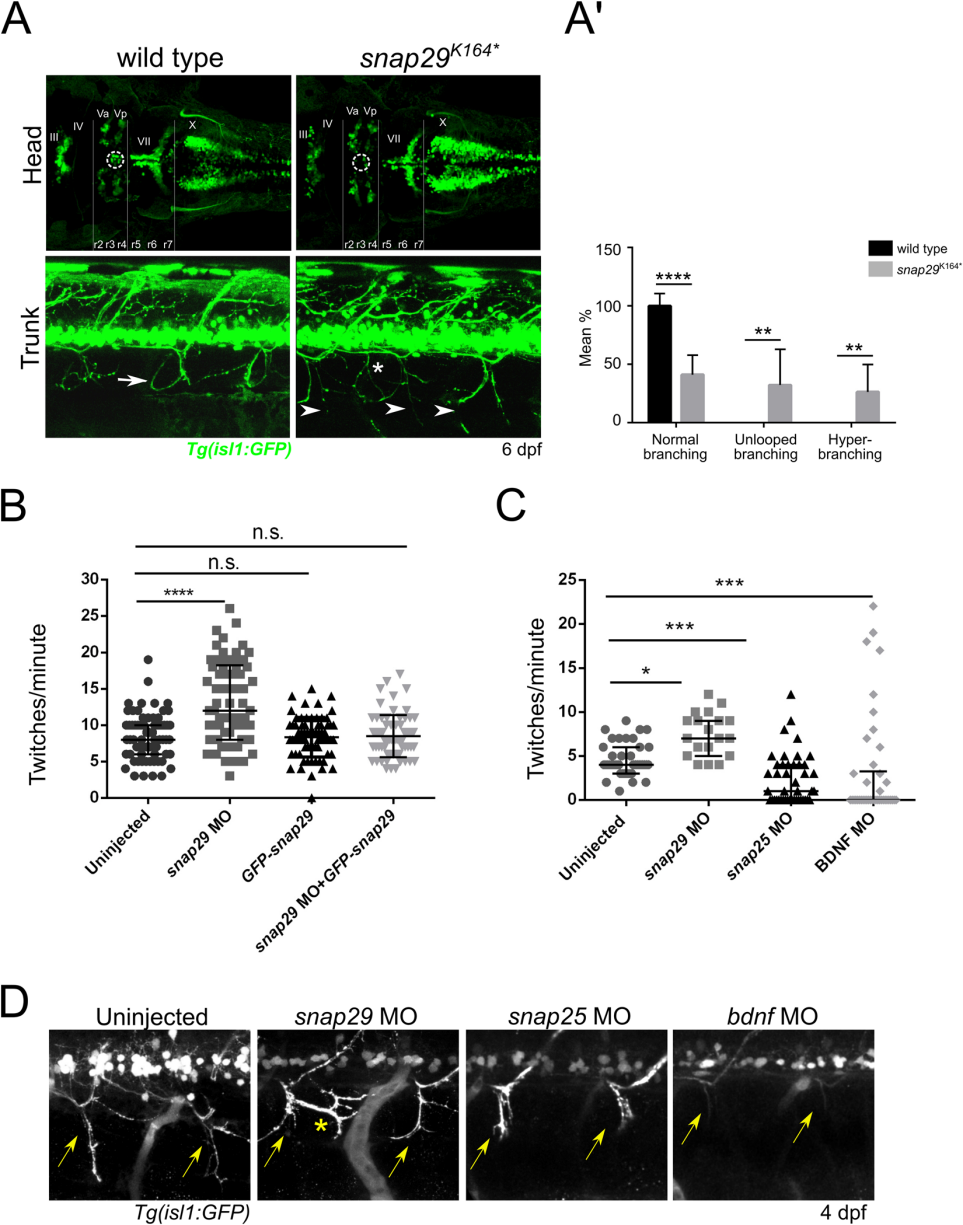
Snap29 impairment causes neuro-developmental defects. (**A**, upper row) Head dorsal views of the motor neuron reporter *Tg(isl1:GFP)* in a wild type and a *snap29*^K164*^ mutant larva at 6 dpf. (r) rhombomere, (III), (IV), (Va) anterior, (Vp) posterior, (VII), (X) cranial nerves. *snap29*^K164*^ mutant larvae lack a group of nuclei (white dashed circles) located between the third and fourth rhombomere (r3, r4). (A, lower row) Lateral views of trunks of *Tg(isl1:GFP)*-expressing wild type and *snap29*^K164*^ mutants at 6 dpf. Compared to the “loop” structures observed in wild type (white arrow), *snap29*^K164*^ mutants present an altered motor neuron projection pattern (arrowheads), and extra branching (white asterisk). (**A’**) Quantification of motor neuron branching in wild type and *snap29*^K164*^ mutant larvae at 4, 5 and 6 dpf. Compared to wild type, *snap29*^K164*^ mutants larvae show a significant increase of unlooped branches and hyperbranching. The bars show means and standard deviations of the percentage of the phenotypic categories. *P*-values were obtained by unpaired t test with Welch’s correction. **P ≤ 0.01, ****P ≤ 0.0001, n = 6–10. (**B**) Quantification of the number of twitches per minute performed respectively by 26 hpf embryos treated as described in figure. *snap29* morphants show an increase in the number of twitches per minute compared to wild type embryos. Co-injection of *snap29* Morpholino with *GFP-snap29* mRNA rescues the increased number of twitches per minute observed in *snap29* morphants, while *GFP-snap29* mRNA injection per se has no effect on twitching frequency. The graph shows medians, 25^th^ and 75^th^ percentiles. *P*-values were obtained by Kruskal-Wallis, ****P ≤ 0.0001, n = 69–73. (**C**) Quantification of the number of twitches as in B. *snap29* morphants show an increase twitches frequency per minute compared to wild type embryos, while both *snap25* and *bdnf* morphants show a significant decrease. The graph shows medians, 25^th^ and 75^th^ percentiles. *P*-values were derived from Kruskal-Wallis, *P ≤ 0.05 ***P ≤ 0.001, n = 20–44. (**D**) Motor neuron projections in 4 dpf *Tg(isl1:GFP)*-expressing uninjected larvae and in *snap29*, *snap25* and *bdnf* morphant larvae. Uninjected embryos show normal motor neuron projection towards the ventral part of the trunk (yellow arrows). *snap29* morphants show less elongated projections (yellow arrows) and extrabranching (yellow asterisk), while both *snap25* and *bdnf* morphants show truncated projections.

To investigate whether neuromotor development is altered in *snap29*^K164*^ mutants, we next used a zebrafish strain derived from crossing the transgenic line *Tg(isl1:GFP)*, in which GFP is expressed in motor neurons^33^, with *snap29*^K164*^ heterozygous fish. By analyzing the brain of 6 dpf larvae derived from the incross of such strain, we observed that homozygous *snap29*^K164*^ mutants lack a group of cells located between the third and fourth rhombomere (Fig. 5A, upper panels, r3, r4). These cells correspond to the trigeminal anterior and posterior motor nuclei (Fig. 5A, upper panels Va and Vp) of neurons that innervate mandibular arch muscles controlling mouth opening^34^. We then analyzed motor neuron projections of *Tg(isl1:GFP) snap29*^K164*^ mutants at the level of developing muscles in the trunk and we detected the presence of an altered branching pattern, compared to the reported “loop” structure^35^ observed in wild type (Fig. 5A, lower panels, white arrows; quantified in 5A’). These results suggest that Snap29 is required to ensure correct neuromuscular system development in zebrafish.

As previously demonstrated by Menelaou *et al*.^36^, during early stages of development spontaneous movements of zebrafish embryos within the chorion (twitches) correlate with correct motor neurons axonal pathfinding. We thus compared spontaneous movements of 26 hpf *snap29* morphants with those of uninjected embryos. We found that twitching frequency increased in *snap29* morphants, compared to uninjected controls, or to embryos injected with *GFP-snap29*, or co-injected with *snap29* MO and *GFP-snap29* (quantified in Fig. 5B; Movie S3–6), indicating that the twitching phenotype is due to loss of Snap29.

In cultured hippocampal neurons of rats, SNAP29 is known to exert a function in synaptic transmission, but unlike the neuronal SNAP family member SNAP25, it appears to act as a negative regulator^17^. Interestingly, we found that while the *snap29*^K164*^ mutant exhibits extra branching within the normal motor neuron projection pattern, *snap25* morphants are reported to show an opposite phenotype, namely a strongly reduced motor neuron arborization^37^. In agreement with this evidence, we observed that in our hands *snap25* morphants show a frequency of ∼1 twitches/minute, which is significantly different from those of *snap29* morphants and uninjected controls (Fig. 5C; Movies S7–9). Similarly, *bdnf* morphants are almost motionless (Fig. 5C; Movies S10). BDNF (Brain-Derived Neutrophic Factor), a secreted molecule belonging to neurotrophin family, together with the nerve growth factors neurotrophin 3,4 and 6^38^, are known to control axonal growth and pathfinding^39^. Consistent with the results of the twitching assay, finally we show that 4 dpf *Tg(isl1:GFP)* expressing *snap29* morphants display altered branching, with extra projections (Fig. 5D, yellow asterisk), while *snap25* and *bdnf* morphants exhibit respectively truncated and thinner projections, not extending ventrally (Fig. 5D, yellow arrows). Overall, these data suggest that perturbation of Snap29 causes an increase in spontaneous movements of embryos, as well as abnormal motor neuron branching. Such phenotypes are for the most part opposite to those observed in *snap25* and *bdnf* morphants, suggesting that Snap29 could act as a negative modulator of motor neuron development.

## Discussion

In this study, we show that zebrafish represents an excellent model organism to study how certain traits of CEDNIK syndrome arise during embryonic development. In particular, we demonstrate for the first time that Snap29 is required to sustain neuromuscular system development and to enable its correct functioning. Both *snap29*^K164*^ and *snap29*^*N171fs*^ mutants, generated respectively by ENU treatment and CRISPR/Cas9 technology, as well as Morpholino treatment, recapitulate the poor life expectancy observed in CEDNIK patients^10^, and show altered skin pigmentation and neuromuscular development. In particular, *snap29*^K164*^ ENU mutants express very low levels of *snap29* mRNA, as is the case of the few CEDNIK patients that have been analyzed molecularly^10^. Importantly, *snap29* paralogs do not appear to be overexpressed in *snap29*^K164*^ ENU mutants, suggesting that their phenotypes are unlikely to be mitigated by functional redundancy. However, it remains to be determined whether wild type levels of *snap29* paralogs might suffice to provide enough proteins to compensate, at least partially, for the loss of *snap29*. The specificity of these phenotypes was confirmed since we were able to completely rescue pigmentation defects, as well as changes in twitching frequency, a phenotype that has been previously associated to altered neurodevelopment. We also partially rescued precocious lethality by injecting mRNA encoding GFP-tagged Snap29. Importantly, pigmentation defects have never been reported neither in human patients, nor in mouse models. However, it might be difficult to evaluate depigmentation considering the presence of ichthyosis and of keratoderma affecting CEDNIK patients^11,12^. Interestingly, melanocytes of the *snap29*^K164*^ mutants do not show migration problems, but they are partially devoid of melanin. The SNARE complex regulating fusion to melanosomes is known to include Syntaxin13 and VAMP7^40^. Since the third SNARE component of this SNARE complex has not yet been identified, based on the pigmentation phenotype, we hypothesize that fusion of enzyme-containing vesicles to melanosomes might depend on Snap29. Thus, we propose that Snap29 could be the third SNARE protein involved in fusions ultimately required for melanin biosynthesis.

Similar to autophagosome accumulation previously observed in *Drosophila Snap29* mutants^4^, extracts of homozygous mutants of *snap29*^K164*^ show increase level of adapter autophagy p62 and of the autophagosome markers LC3II, a phenotype characteristic of impaired autophagy. Whether and how alteration of autophagy contributes to CEDNIK pathogenesis is unclear. Interestingly, mutations in human *EPG5* (*ectopic P-granules autophagy protein 5*) cause a severe neurodevelopmental disease called Vici syndrome, which shares with CEDNIK many clinical manifestations in pediatric patients, such as microcephaly, brain development abnormalities, atrophy of the retina and muscle hypotonia^41^. Importantly, EPG5 was recently reported to regulate the autophagosome-lysosome fusion step^42^. Moreover, similarly to *snap29*^K164*^ mutant zebrafish, Vici patients exhibit hypopigmentation and muscle biopsies from Vici patients show accumulation of p62. Thus, based on the similarity with Vici syndrome, it is likely that aspects of CEDNIK pathogenesis might also derive from impaired autophagic clearance.

Other congenital neurodevelopment syndromes like Roberts syndrome and Primary microcephaly (MCPH) are caused by mutations in genes that regulate cell division^43,44^. Importantly, a very recent work demonstrated that Snap29 is required to prevent cell division defects and apoptosis in both *Drosophila* and human cells^9^. Consistent with this, *snap29*^K164*^ mutants present a high number of apoptotic cells during early development. It is thus possible that the massive cell death occurring in the head of *snap29*^K164*^ mutants at 19 hpf and 3 dpf could contribute to the microcephaly that becomes apparent at 7 dpf, as well as to reduction in thickness of the skin peridermal layer. In such scenario, neuronal and peridermal proliferation might be reduced by early loss of cells that might behave as progenitors, with surviving cells incompletely able to compensate in mutant animals. Consistent with this, we have observed that at 19 hpf the amounts of proliferating cells in mutant animals is not increased when compared to controls, suggesting that the increased apoptosis in mutants is not compensated by an increase in proliferation.

CEDNIK patients suffer also from neurogenic atrophy, which is loss of muscle tone caused by wasting of nerves controlling muscles^12^. Similarly, *snap29*^K164*^ mutants show defective muscle fibers organization, which could be the cause of the observed reduced larval motility. Their muscles also contain mitochondria enveloped in a double-membrane organelle. Recent reports pointed out that Snap29, together with Syntaxin17 and VAMP7, is required for the fusion of mitochondrial-derived vesicles (MDVs) with endolysosomal compartments to promote eventually mitochondrial degradation in lysosomes^45,46^. Thus, mitochondria efficiency in supporting muscle activities could be affected by a failure in clearance of mitochondrially-derived vesicles.

In addition to be due to potentially impaired muscle functionality, reduced touch-evoked responses of *snap29*^K164*^ mutant larvae might echo nervous system manifestations of CEDNIK patients, which include psychomotor retardation^12^. In addition, absence of trigeminal motor neurons, which control mandibular arch muscles and mouth opening^33^ in mutant fish, suggests a further cause for lack of swim bladder inflation and feeding impairment, the latter phenotype echoing the inability to feed of CEDNIK infants^31^.

Whichever the case, the analysis of motor neurons innervating skeletal muscles of the trunk of mutant larvae, revealed abnormal axon projections branching at different developmental stages. Importantly, SNAP25 and SNAP47 regulate neuronal circuit development and axon branching by mediating the membrane fusion events required for release of neurotransmitters and of the brain-derived neurotrophic factor (BDNF), which is normally stored in dense core vesicles (DCVs)^47–49^. Considering that overexpression of Snap29 in Snap25 KO neurons is able to restore DVCs release^50^, it is possible that the lack of Snap29 results in uncontrolled release of neurotransmitters and BDNF, eventually affecting axon branching. This hypothesis is supported by the increase in spontaneous twitches within the chorion observed in 26 hpf *snap29* morphants compared to uninjected embryos, a specific phenotype which is rescued by ectopic expression of GFP-Snap29. In contrast, as previously reported^37,51^, we observed an opposite phenotype in *snap25* and *bdnf* morphants, consisting in decreased twitches per minute. Spontaneous movements have been shown as required for normal motor neuron development and axonal branching. Indeed, their decrease, induced in 24 hpf embryos by the treatment with the anesthetic tricaine, also determines motorneuronal axonal pathfinding defects^36^. Accordingly, *snap29* morphants present a motorneuron hyperbranching (as observed for of *snap29*^K164*^ mutant), while *snap25* and *bdnf* morphants show, respectively, truncated and thinner projections compared to uninjected embryos. Overall, we surmise that Snap29 might be fundamental, possibly, for normal neuromuscular development as a regulator of membrane fusion, which is controlled by a finely-tuned release of BDNF.

In summary, our zebrafish mutant analysis provides the base for future study of the pathogenesis of CEDNIK, especially regarding the unexplored neuromotor features of the syndrome. Mutant larvae could be also used to test new compounds that might mitigate the consequences of the most deleterious traits of the disease.

## Materials and Methods

### Zebrafish strains

Adult zebrafish were maintained in a commercial system (Aquatic Habitat) at a water temperature of 28.5 °C, pH 7 and conductivity 500 μS. Zebrafish embryos and larvae not older than 5 dpf are maintained at 28 °C in E3 water (50 mM NaCl, 0.17 mM KCl, 0.33 mM CaCl, 0.33 mM MgSO4, 0.05% methylene blue). Zebrafish strains used in this study are AB (referred to as wild type), *sa13359* obtained from European Zebrafish Resource Center referred to as *snap29*^K164*^, *snap29*^*N171fs*^ generated by CRISPR/Cas9 technology (see details below), and *Tg*(*isl1:GFP)*^33^. All the strains were maintained and bred according to the national guidelines (Italian decree ‘4 March 2014, n.26’). All experimental procedures were approved by the FIRC Institute of Molecular Oncology Institutional Animal Care and Use Committee and Italian Ministry of Health.

### *In situ* hybridization

Zebrafish embryos were fixed in 4% PFA at 4 °C O/N. Digoxigenin (DIG)-labeled antisense probes were synthesized with DIG RNA labelling MIX kit (Roche) using a DNA template amplified from cDNA using specific primers: *snap29* T3 5′-taatacgactcactatagggagaATGTCTGCCTACCCCAAATC-3′, *snap29* T7 5′-attaaccctcactaaagggagaACATCTCATCCAGGTTTCT-3′. After hybridization, detection was performed with anti-DIG antibody coupled to alkaline phosphatase (Roche) and specimen were imaged with Olympus SZX12 stereomicroscope.

### CRISPR/Cas9 *snap29* mutagenesis in zebrafish and *sa13359* genotyping

The short guide (sg) RNA AGGCCAGTCATCCAAACCTCAGG targeting the exon 4 of *snap29* zebrafish gene, was synthetized *in vitro* starting from the annealing of oligonucleotide 1 5′-TAGGCCAGTCATCCAAACCTC-3′ and oligonucleotide 2 5′-AAACGAGGTTTGGATGACTGG-3′ previously diluted in annealing buffer (10 mM Tris, pH 7.5–8, 50 mM NaCl, 1 mM EDTA) to reach the final concentration of 100 mM. After having mixed the oligos together in equal proportion, they were heated at 95 °C for 3–5 minutes and cooled at room temperature for 60 minutes. The resulting sgRNA was cloned in a DR274 vector (Addgene) and RNA *in vitro* transcription was performed with a standard kit (MEGAscript T7 Transcription Kit, Ambion) using the linearized DR274 plasmid as template. The sgRNA was injected together with the Cas9 purified protein (prepared by IFOM Biochemistry unit) in zebrafish embryos and mosaic animals were obtained. To monitor the presence of mutations, mosaic animals were subjected to the mismatch sensitive endonuclease T7 (T7E) (NEB) assay. Briefly, genomic DNA (gDNA) was extracted from caudal fin biopsies (fin clip) of adult animals, and a fragment of 500 bp containing the sgRNA complementary region was amplified by PCR with the specific primers forward 5′-ACCCCAAATCCCACAATCCT-3′ and reverse 5′-GGCGTAACTAGGTTCATTAGGG-3′. The resulting PCR products were subjected to denaturing/annealing steps (95 °C 2 min, −2 °C/s to 85 °C, −0.1 °C/s to 25 °C, 16 °C) and digested by T7E.

To isolate potential founders bearing mutations in the germline, mosaic animals were outcrossed to wild type (AB) animals. A pool of 10 embryos derived from each single cross were subjected to T7E assay. To establish *snap29* mutant strains, founder animals were then crossed with AB animals. When heterozygous offspring reached adulthood by fin clip, gDNA from 20 animals was extracted, amplified with the primers above, and sequenced (Cogentech Sequencing Facility).

gDNA extracted from heterozygous animals obtained from the outcross of the *snap29* mutant strain *sa13359* (generated with ENU at Sanger Institute)^52^ with AB animals was sequenced using the same procedure described above.

### Generation and injection of *GFP-snap29*

To generate the GFP*-snap29* plasmid used for *snap29*^K164*^ mutant rescue, the zebrafish *snap29* coding sequence was amplified using as template 24 hpf embryo cDNA and as primers BglII-Snap29 forward 5′-TCGAGAAGATCTATGTCTGCCTACCCCAAATCCC-3′ and XhoI-Snap29 reverse 5′-ATCGCCCTCGAGCTATTTAAGGCTTTTGAGCTG-3′. Both the PCR product and the pEGFP plasmid (Addgene) were digested using BglII and XhoI restriction enzymes (New England Biolab, NEB), purified using QIAquick Gel Extraction Kit protocol (QIAGEN) and subjected to ligation with T4 DNA ligase (NEB) according with manufacturer instructions. The plasmid obtained was used as a template for a second PCR using as primers BamHI-GFP forward 5′-ATCGCGGGATCCATGTGAGCAAGGGCGAGG-3′ and XhoI-Snap29 reverse. Both PCR product and pCS2 plasmid (Addgene) were digested with BamHI and XhoI restriction enzymes, purified and subjected to ligation with T4 DNA ligase (NEB).

pCS2 *GFP-snap29* was used as templates to synthesize mRNAs using MAXIscript SP6 Transcription Kit (Ambion). 200 pg of mRNA were injected in one-cell stage embryos.

### RNA extraction from zebrafish, cDNA synthesis, qPCR and RT-PCR

Wild type zebrafish larvae (AB strain) were collected at 96 hpf and RNA was extracted using TRIZOL Reagent (Invitrogen) and RNAse Mini kit (QIAGEN). To avoid genomic DNA contamination, samples were digested with RQ1 RNase-Free DNase (Promega). The cDNA was retrotranscribed from 1 μg of RNA using SuperScript VILO cDNA Synthesis kit (Invitrogen), according to manufacturer instructions. 500 ng of cDNA were used as template for real time PCR (qPCR) reactions performed by Cogentech Real Time Quantitative PCR service using the following primers: *snap29* forward 5′-ATCTGGGACAACTTGGGCAACT-3′, *snap29* reverse 5′-GAGCGTCCAGAGAAATGTCC-3′, GAPDH forward 5′-TCAGTCCACTCACACCAAGTG-3′, GAPDH reverse 5′-CGACCGAATCCGTTAATACC-3′, *snap23.1* forward 5′-TGTATCCAGCCAACCGACTG-3′, *snap23.1* reverse 5′-GAAGTTTGTTGGCTCGCTGG-3′, *snap23.2* forward 5′-AATCCCAGTCCAGCGTGATG-3′, *snap23.2* reverse 5′-GTGGGCTTCAGTCTGGAACA, *snap25a* forward 5′-AGCAGCTCAGTCCCTACAGA-3′, *snap25a* reverse 5′-TGGTCCATTCCCTCCTCGAT-3′, *snap25b* forward 5′-GCTGGGCGATGAATCTTTGG-3′, *snap25b* reverse 5′-CCCGACCTGCTCCAAATTCT-3′, *snap47* forward 5′-CTTATCTCGCACCACCCTCC-3′, *snap47* reverse 5′-CAGACTTGGCCTCCTGATGG-3′.

### Genomic DNA extraction from zebrafish embryos

24 hpf embryos were dechorionated with 1 mg/ml Pronase (Sigma-Aldrich) for 15 minutes at 37 °C. To dechorionate 50 embryos, 50 μl of lysis buffer (Tris-HCl 10 mM pH 8.0, EDTA 1 mM, 0.3% Tween, 0.3% NP40) were added. Incubation lasted 10 minutes at 98 °C followed by the ice cooling. 5 μl of Proteinase K 10 mg/ml (Sigma-Aldrich) were added and embryos were incubated at 55 °C O/N. The second day, 145 μl of sterile water were added, followed by 20 μl of Sodium Acetate and 200 μl of Phenol. Samples were mixed by inverting them and centrifuged at 13000 rpm for 1 minute. Supernatant was collected and precipitated O/N with 100% ethanol at −20 °C. The third day, samples were centrifuged for 30 minutes at 4 °C and recovered pellets were washed with 75% ethanol, centrifuged again for 5 minutes and resuspended in 20 μl of DNAse-free water.

### Hematoxylin and eosin staining and immunostaining on paraffin sections

Larvae were fixed O/N at 4 °C in 4% PFA diluted in PBS and positioned in a 7 × 7 × 6 mm plastic base-molds (Kaltek) containing 1.2% low-melting agarose in PBS. Before agarose solidification, larvae were correctly oriented. After agarose block solidification, larvae were removed from the base mold and immersed in 70% ethanol. After dehydration, agarose blocks were subjected to paraffin embedding by Leica ASP300 S Fully Enclosed Tissue Processor and 5 μm thick sections were cut using a manual rotatory microtome (Leica).

Sections were deparaffinized in histolemon for 5 minutes, hydrated with 100%, 95% and 80% ethanol, respectively for 5 minutes each for 3 times, and finally rinsed with distilled water.

Sections were stained with Harris hematoxilin solution for 2 minutes, washed in running water for 5 minutes, counterstained with Eosin-Y solution for 7 seconds and washed in running tap water for 5 minutes. Sections were dehydrated with 95% ethanol and 100% ethanol for 5 minutes two times. Then, they were cleared two times with xylene for 5 minutes and mounted on a glass slide. Sections were finally imaged using a Nikon Eclipse 9i microscope, respectively with 20× and 100× objectives.

For immunostaining, sections were incubated in sodium citrate buffer (2.94 mg/ml tri-sodium citrate pH 6, 0.05% Tween 20) at 95 °C for 45 minutes and cooled at RT for 1 hour under chemical hood. Sections were then incubated in blocking solution (2% fetal bovine serum, 2 g bovine serum albumin, 0.05% Tween 20 in PBS 1X adjusted at 7.2 pH) for 1 hour at RT followed by primary antibodies diluted in blocking solution O/N. Samples were rinsed in PBS 1X three times for 5 minutes. Secondary antibodies diluted in PBS 1X were added and incubated for 1 hour and then washed three times with PBS 1X. Slides were incubated with DAPI for 5 minutes at RT, rinsed in PBS 1X three times for 5 minutes and mounted on a glass slide in 50% glycerol. The following primary antibodies were used: rabbit anti-p62 1:1000 (Enzo Life Science), mouse anti-pH3 1:1000 (Abcam), rabbit anti-cleaved Caspase 3 1:100 (Cell Signaling). Alexa fluor 488 and 647 conjugated (Invitrogen) were used as secondary antibodies.

### Zebrafish whole-mount immunostaining

Embryos or larvae were fixed O/N at 4 °C with 4% PFA diluted in PBS 1X and rinsed 3 times with PBS 1X. Embryos older than 24 hpf were treated with 0.25% trypsin (Sigma-Aldrich) at RT for a range of time between 2 minutes (for 24 hpf embryos) up to 60 minute (for 5 dpf larvae). Samples were then rinsed 3 times for 5 minutes with washing buffer (1% Triton-X100, 0.2% DMSO in PBS 1X) and incubated for at least 1 hour in blocking buffer (0.1% Triton X-100, 1% DMSO, 5% normal goat serum in PBS 1X) on a shaker. Subsequently, embryos were incubated with primary antibodies diluted in blocking buffer O/N at 4 °C. The following day, samples were rinsed rapidly twice with washing buffer and at least 3 washes of 1–2 hours each with washing buffer were performed. Samples were incubated in blocking buffer for 30 minutes followed by secondary antibodies diluted in blocking buffer O/N at 4 °C. The final day, samples were rapidly rinsed 2 times with washing buffer and two washes of 5 minutes each with PBS 1X were performed. Samples were incubated 10 minutes with DAPI, rapidly rinsed with PBS 1X and mounted on a glass slide in 85% glycerol.

The following primary antibodies were used: mouse anti-Myosin heavy chain (all-Myo) 1:20 (Developmental Studies Hybridoma Bank), rabbit anti-cleaved Caspase 3 1:200 (Cell Signaling), chicken anti-GFP 1:1000 (Abcam), mouse anti-pH3 1:1000 (Abcam). Alexa fluor 488, 543 and 647 (Invitrogen) were used as secondary antibodies.

### Touch-evoked response assay

AB and *snap29*^K164*^ mutant 6 dpf larvae were mechanically stimulated with a plastic tip and recorded for 1 minute with a NIKON DS-5MC digital camera, mounted on a NIKON SMZ-1500 stereomicroscope. 6 larvae for each condition were used and only the first 5 stimuli were considered for quantification. Statistical analysis was performed with Prism Software.

### Rhodamine Dextran food preparation

Rhodamin-dextran was mixed with two different larval foods commonly used for the larval feeding. In particular, 100 mg of “Larval AP100 food” (microparticles size < 100 microns), 100 mg of JBL “Novo Tom” lyophilized artemia and 40 μl of 20 mg/ml Rhodamin-Dextran 10000 MW (Invitrogen) were added to 360 μl of Milli-Q water. The mixture was dropped on a glass slide and dried O/N at RT, protected from light exposure, then reduced to a fine powder with a pestle and administered to larvae by dissolving it on the water surface.

### Morpholino injections

Zebrafish embryos were microinjected with an Olympus SZX9 and a Picospritzer III microinjector (Parker Instrumentation). Injection mixes were composed of Danieau solution 1X (NaCl 58 mM, KCl 0.7 mM, MgSO_4_ 0.4 mM, Ca(NO_3_)_2_ 0.6 mM, HEPES 5.0 mM, pH 7.6), Phenol red 0.1% and Morpholino (MO) antisense oligos (Gene Tools). Each embryo was injected respectively with 1.7 ng of splice blocking *snap29* MO^14^, 1.7 ng of splice-blocking *snap29* together with 200 pg *GFP-snap29* mRNA for the rescue, 3 ng of 5′-UTR *snap25 a,b* MO, or 3 ng of ATG *bdnf* MO^51^.

### Twitching assay

Spontaneous motility assay (twitching assay) was performed using 26 hpf embryos in 3.5 mm Petri dish by recording embryos for 1 minute with a NIKON DS-5MC digital camera mounted on a NIKON SMZ-1500 stereomicroscope.

### Electron microscopy

7 dpf larvae were fixed for 2 hours at RT with a mixture of 4% paraformaldehyde and 2.5% glutaraldehyde (EMS, USA) in 0.2 M sodium cacodylate pH 7.2, followed by 6 washes in 0.2 sodium cacodylate pH 7.2 at RT. Samples were then incubated in a 1:1 mixture of 2% osmium tetraoxide and 3% potassium ferrocyanide for 1 hour at RT followed by 6 times rinsing in 0.2 M cacodylate buffer. Samples were sequentially treated with 0.3% thiocarbohydrazide in 0.2 M cacodylate buffer for 10 minutes and 1% OsO4 in 0.2 M cacodylate buffer (pH 6.9) for 30 minutes. Then, samples were rinsed with 0.1 M sodium cacodylate (pH 6.9) buffer until all traces of the yellow osmium fixative had been removed. Then they were washed in de-ionized water, treated with 1% uranyl acetate in water for 1 h and washed in water again. Samples were subsequently subjected to dehydratation in ethanol followed by acetone, and embedded in Epoxy resin at RT, which was polymerized for at least 72 hours in a 60 °C oven. Embedded samples were sectioned with a diamond knife (Diatome, Switzerland) using ultramicrotome (Leica EM UC7; Leica Microsystems, Vienna). Sections were analyzed with a Tecnai 20 High Voltage EM (FEI, Thermo Fisher Scientific, Eindhoven, The Netherlands) operating at 200 kV^53^. Electron microscopic examination was performed as previously described^54–56^.

### Measurements

Quantifications were performed with Fiji. Prism GraphPad was used for statistical analyses. Choice of statistical tests and sample size are detailed in figure legends. *P*-values are as follows: *P ≤ 0.05, **P ≤ 0.01, ***P ≤ 0.001, ****P ≤ 0.0001.

Quantification of head and trunk area were performed by manually drawing a region of interest (ROI) and by measuring the number of enclosed pixels.

The number of apoptotic and proliferative cells in each sample was determined by manually counting the number of apoptotic cells on maximum projection images of 0.5 μm z-stacks. The absolute number was normalized by the area, which was manually drawn around DAPI signals.

Myofibrils profile length was determined by manually drawing the profile of superficial myofibrils stained with the anti-Myosin antibody.

Twitching assay was quantified by drawing a ROI around each embryo and by counting manually the number of spontaneous movements per minute. ROI files for each experiment are available upon request.

## Supplementary information

supplementary data

movieS1

movieS2

movieS3

movieS4

movieS5

movieS6

movieS7

movieS8

movieS9

movieS10
