# Supplementary material for: A genetic model of CEDNIK syndrome in zebrafish highlights the role of the SNARE protein Snap29 in neuromotor and epidermal development
Source: Sci Rep. 2019 Feb 4;9:1211. doi: 10.1038/s41598-018-37780-4 (PMC6361908; doi:10.1038/s41598-018-37780-4)
Supplement: Supplementary file 1 — supplementary data [file 41598_2018_37780_MOESM1_ESM.pdf]

Supplementary figures

A genetic model of CEDNIK syndrome in zebrafish highlights the role of the SNARE protein Snap29 in neuro-motor and epidermal development

Valeria Mastrodonato[1,3], Galina Beznoussenko[1], Alexandre Mironov[1], Laura Ferrari[2], Gianluca Deflorian[1*] and Thomas Vaccari[3*]

Fig. S1

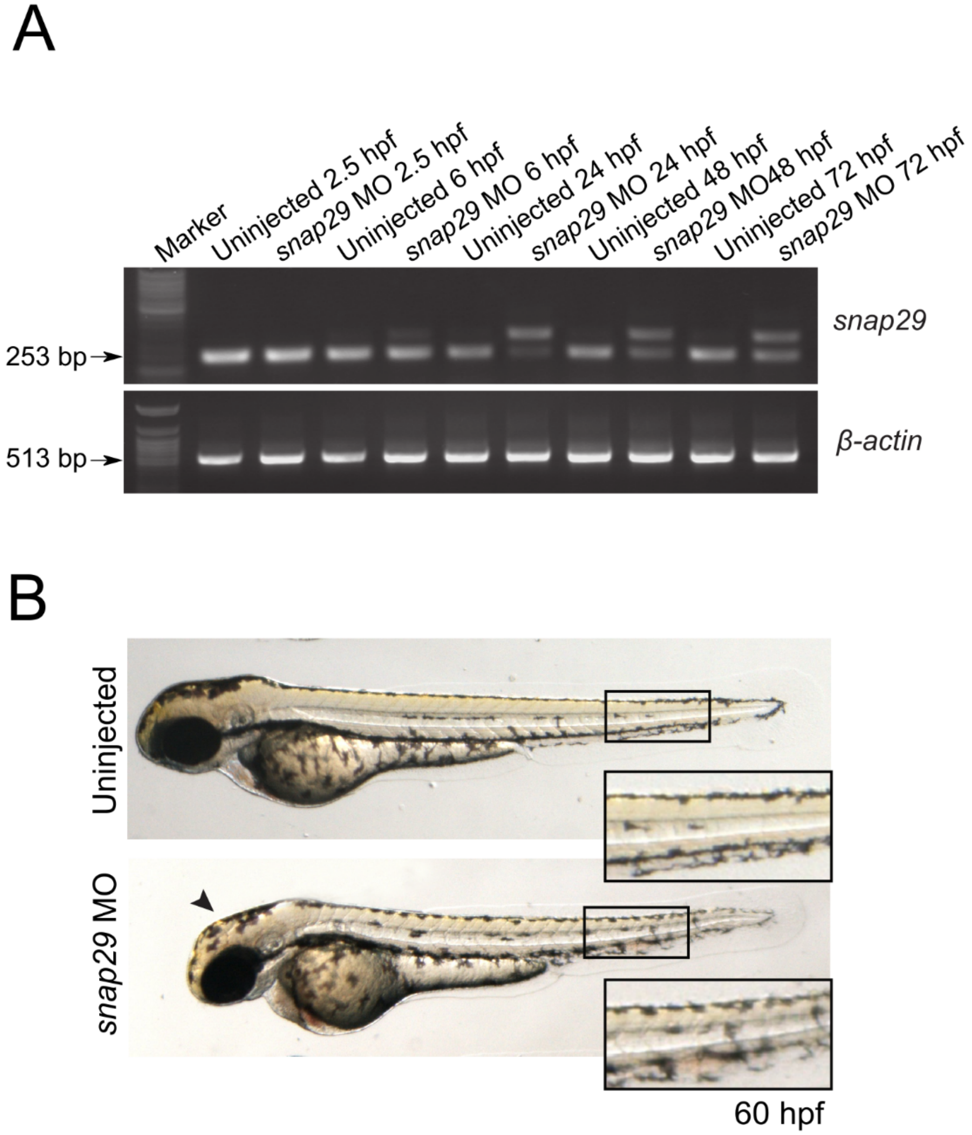
A
Marker
Uninjected 2.5 hpf
snap29 MO 2.5 hpf
Uninjected 6 hpf
snap29 MO 6 hpf
Uninjected 24 hpf
snap29 MO 24 hpf
Uninjected 48 hpf
snap29 MO48 hpf
Uninjected 72 hpf
snap29 MO 72 hpf
snap29
253 bp
β-actin
513 bp
B
Uninjected
snap29 MO
60 hpf

Fig. S2

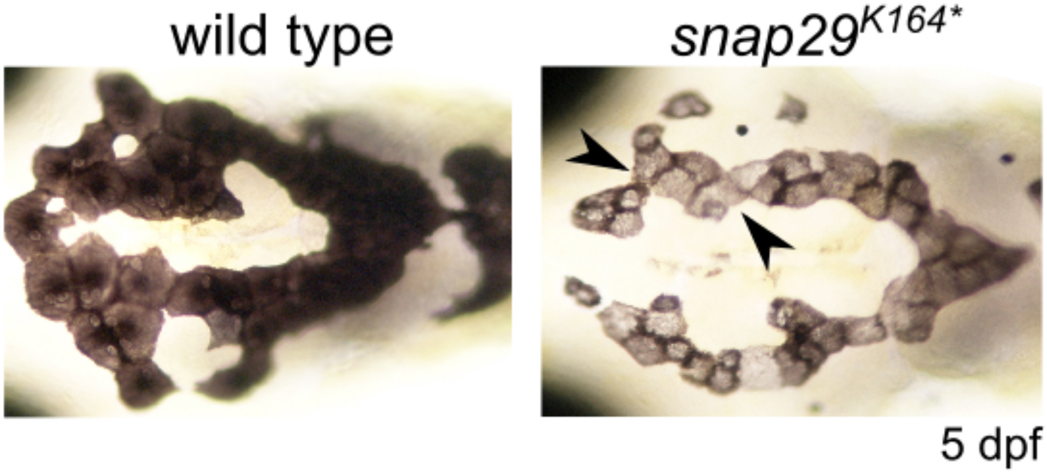

Fig. S3

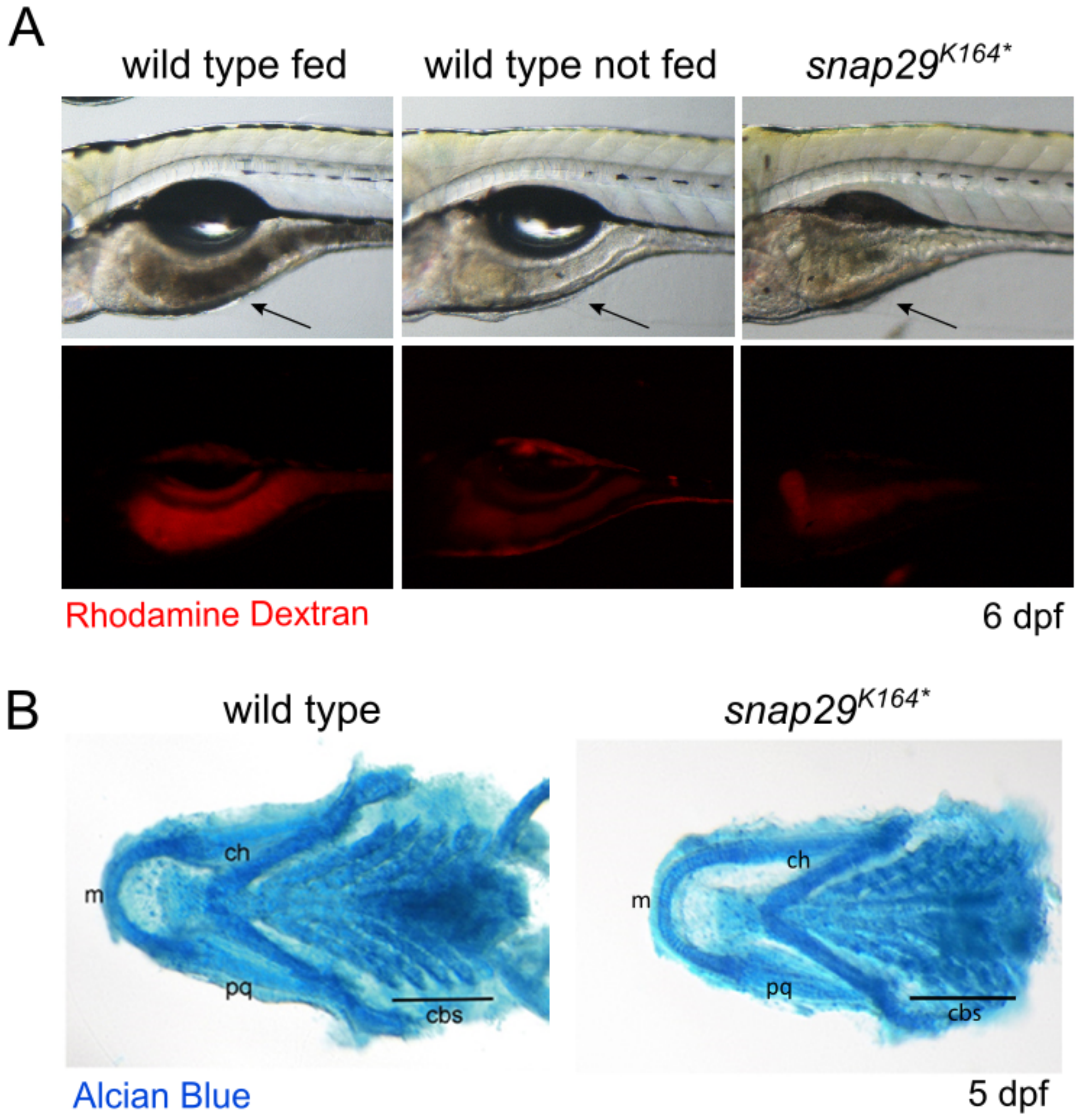
A
wild type fed
wild type not fed
snap29K164*
Rhodamine Dextran
6 dpf
B
wild type
snap29K164*
ch
m
pq
cbs
ch
m
pq
cbs
Alcian Blue
5 dpf

Fig. S4

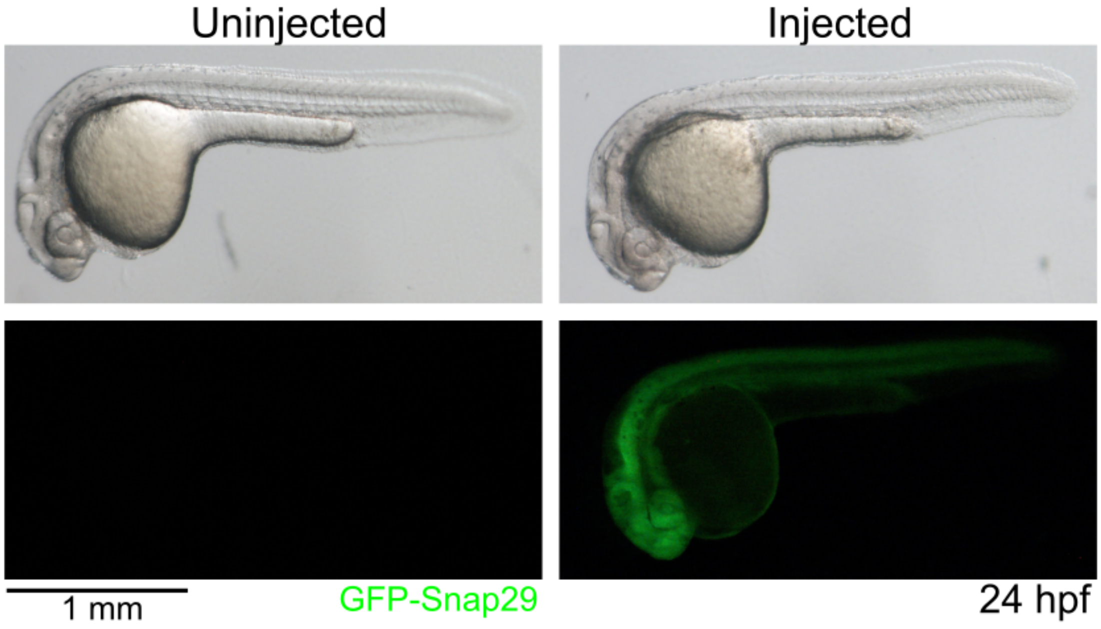

Fig. S5

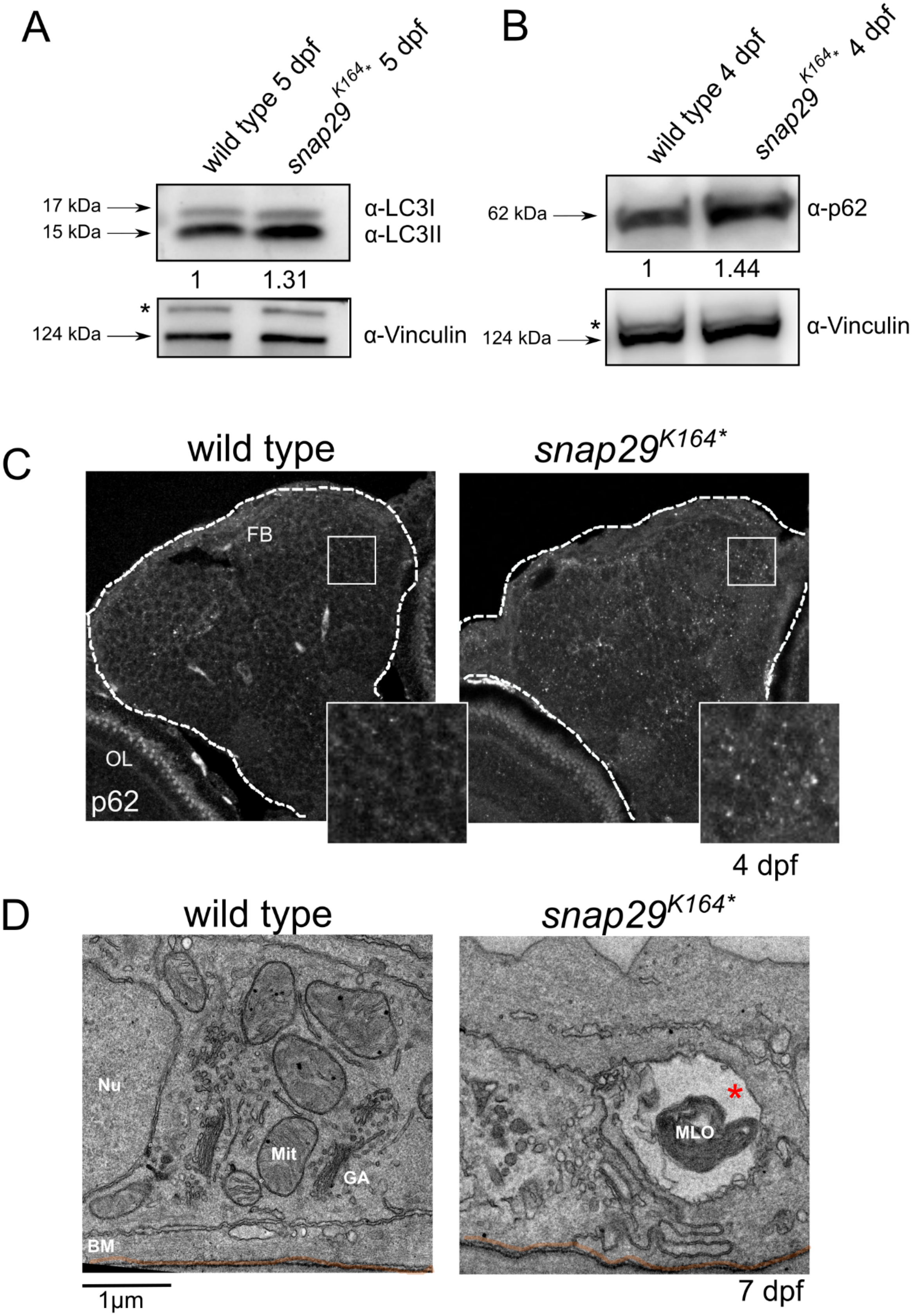
A
wild type 5 dpf
snap29K164* 5 dpf
17 kDa
15 kDa
α-LC3I
α-LC3II
1
1.31
*
124 kDa
α-Vinculin
B
wild type 4 dpf
snap29K164* 4 dpf
62 kDa
α-p62
1
1.44
*
124 kDa
α-Vinculin
C
wild type
snap29K164*
FB
OL
p62
4 dpf
D
wild type
snap29K164*
Nu
Mit
GA
BM
MLO
*
1µm
7 dpf

Fig. S6

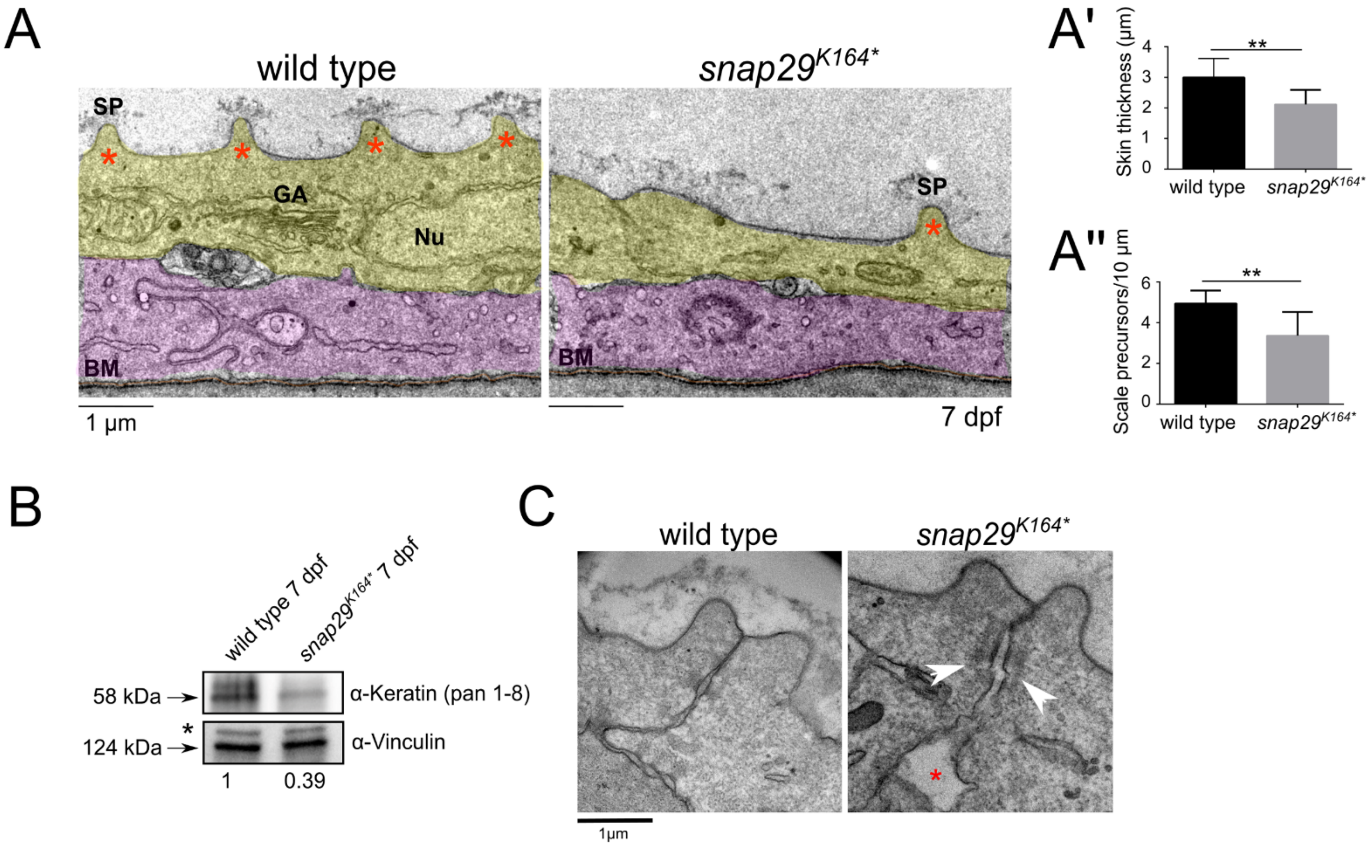
A
wild type
snap29K164*
SP
GA
Nu
BM
SP
1 µm
7 dpf
A'
Skin thickness (µm)
**
wild type
snap29K164*
A''
Scale precursors/10 µm
**
wild type
snap29K164*
B
wild type 7 dpf
snap29K164* 7 dpf
58 kDa
α-Keratin (pan 1-8)
124 kDa
α-Vinculin
1
0.39
C
wild type
snap29K164*
1µm

Fig. S7

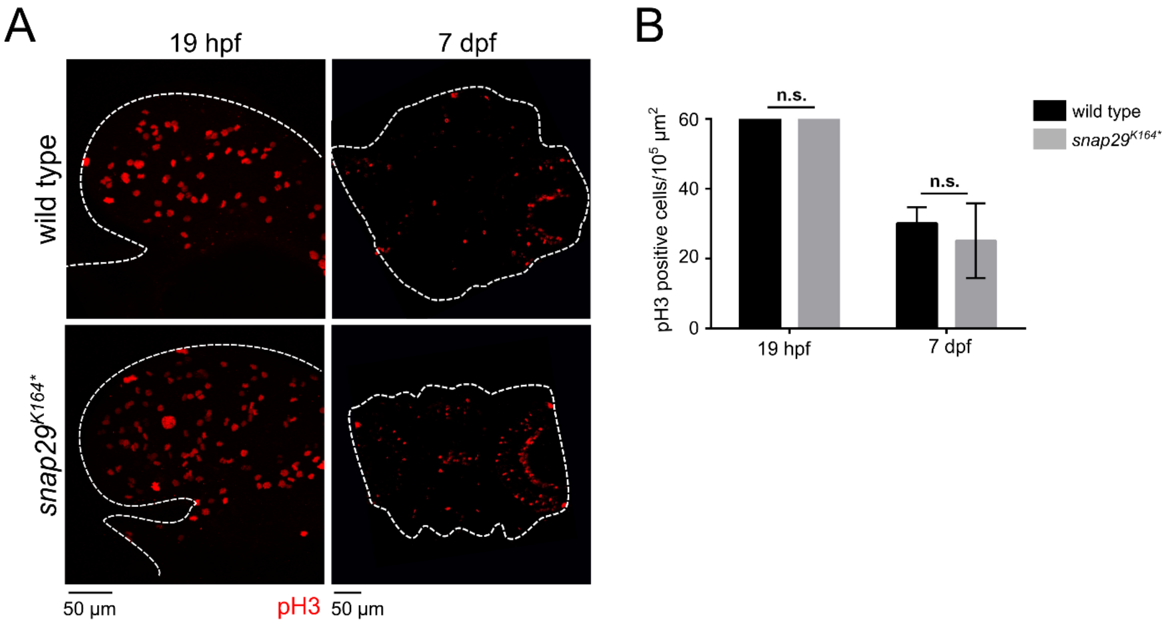
A
19 hpf
7 dpf
wild type
snap29K164*
50 μm
pH3
50 μm
B
n.s.
n.s.
pH3 positive cells/10^5 μm^2
60
40
20
0
19 hpf
7 dpf
wild type
snap29K164*

Fig. S8

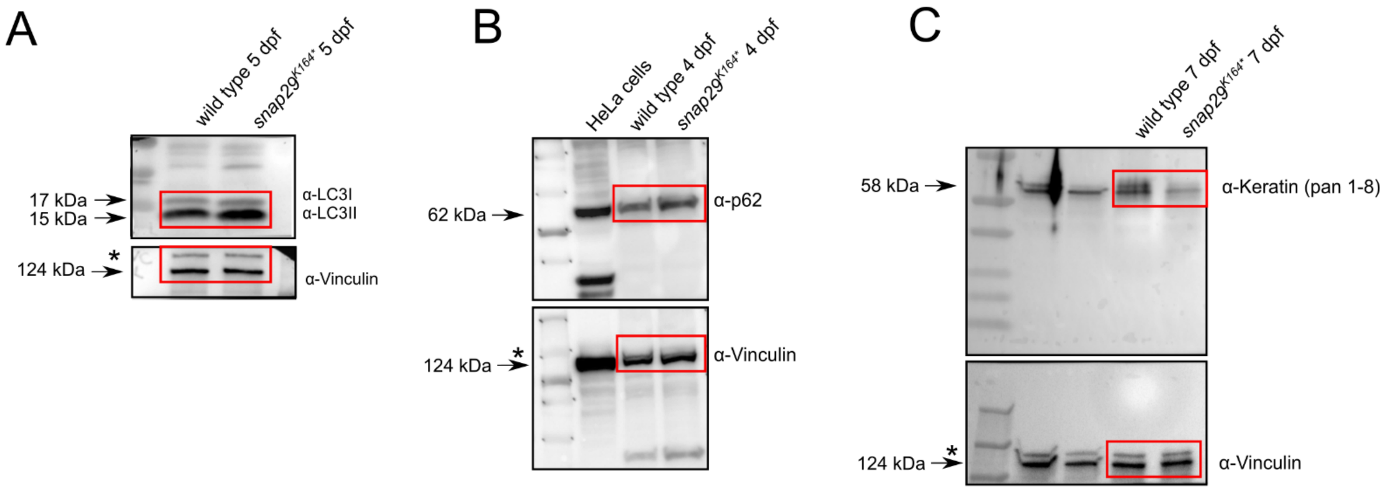
A
wild type 5 dpf
snap2g K164* 5 dpf
17 kDa
15 kDa
α-LC3I
α-LC3II
*
124 kDa
α-Vinculin
B
HeLa cells
wild type 4 dpf
snap2g K164* 4 dpf
62 kDa
α-p62
*
124 kDa
α-Vinculin
C
wild type 7 dpf
snap2g K164* 7 dpf
58 kDa
α-Keratin (pan 1-8)
*
124 kDa
α-Vinculin

Supplementary materials and methods

Reverse Transcriptase-PCR (RT-PCR)

RNA was extracted and retrotranscribed as described in Materials and Methods section. 200 ng of cDNA was used as template for Reverse Transcriptase-PCR (RT-PCR) using the following primers: *snap29* forward 5'-TTCTGCTGCTCTTGATAACGGCT-3', *snap29* reverse 5'-TTTAAGGCTTTTGAGCTGCCGGT-3', *β-actin* forward 5'-ACCTGATGAAGATCCTGACC-3', *β-actin* reverse 5'-TGCTAATCCTCATCTGCTGG-3' [14].

Protein extraction and Western blots

Deyolked zebrafish larvae were washed with PBS 1X, centrifuged at 3000 g and homogenized with Laemmli buffer (20% SDS, 50% Glycerol, 0.01% β-Mercapto-ethanol, Tris-HCl 1 M pH 6.8) using a pestle and an insulin needle. Samples were incubated at 98°C and cooled on ice for 3 times. Extracts were centrifuged at 13400 rpm for 5 minutes, collected in a clean tube and quantified by BiCinchoninic acid Assay (BCA Assay, ThermoScientific). For Western blot, proteins were denatured with Laemmli Buffer 2X (6.25 mM Tris-HCl pH 6.8, 1% glycerol, 2% SDS, 2% β-mercaptoethanol, 0.0012% bromophenol blue) and boiled for 5 minutes. Lysates were run on 4-20% Mini-PROTEAN Precast Gel (Biorad). Immunoblots signals were detected with HRP Chemiluminescent substrate (Thermo Fischer) using Chemidoc (Biorad). Primary antibodies used were rabbit anti-LC3 1:1000 (Thermo Fischer), rabbit anti-p62 (Enzo-LifeScience) 1:1000, mouse anti-Vinculin 1:5000 (Amersham), rabbit anti-Keratin pan 1-8 (Progen) 1:500. Densiometric quantification was performed with ImageLab and anti-Vinculin signal was used as normalizer.

Alcian blue staining of larval cartilages

6 dpf zebrafish larvae were fixed with 4% PFA O/N at 4°C. Samples were washed two times with sterile water and incubated in Alcian Blue solution pH 2.5 (Bioptica) O/N at RT. After staining, larvae were washed 3 times with sterile water and incubated in 3% H_2O_2 solution for 30 minutes at RT. Larvae were washed with sterile water, gradually dehydrated with ethanol and equilibrated in a solution of 85% glycerol in PBS1X. Splanchnocrania were manually dissected from the heads and imaged with a NIKON DS-5MC digital camera mounted on a NIKON SMZ-1500 stereomicroscope.

Supplementary figure legends

Figure S1.

(A) RT-PCR performed to assess *snap29* transcript level using cDNA from embryos uninjected or injected with a splicing block Morpholino against *snap29* (SB MO *snap29*) at the indicated hours post fertilization (hpf). *β-actin* was used as normalizer. (B) 60 hpf uninjected embryos or embryos injected with SB *snap29* MO. Morphants display lighter pigmentation at the level of the head (arrowheads) and misaligned melanocytes in the tail contour (high magnification in insets), compared to uninjected embryos.

Figure S2. *snap29* mutants show depigmented melanocytes

Bright-field dorsal views of the head of a wild type and a *snap29*^{K164*} mutant animal at 5 dpf. Arrowheads indicate lightly pigmented melanocytes in *snap29*^{K164*} mutant, compared to wild type.

Figure S3. Impaired feeding in *snap29* mutants

(A) Lateral views of 6 dpf larvae in which guts are indicated by an arrow. Larvae were fed Rhodamine Dextran-containing food that allow detecting the presence of food in the gut. The gut of a *snap29*^{K164*} mutant animal appears as empty as that of unfed wild type larvae, when compared to fed ones. (B) Ventral views of the splanchnocranium of 5 dpf larvae. Cartilage staining with Alcian Blue did not highlight any difference in organization and/or differentiation between wild type and *snap29*^{K164*} mutants. m: Meckel's cartilages, ch: ceratohyal, pq: palatoquadrate, cbs: ceratobranchials.

Figure S4. *GFP-snap29* expression in zebrafish

24 hpf embryos uninjected and injected at single-cell stage with *GFP-snap29* mRNA. Bright-field and fluorescent images are shown, indicating that the mRNA is translated into ectopic protein ubiquitously.

Figure S5. *snap29* mutants display impaired autophagy

(A) Detection of zebrafish LC3 protein by Western blot in extracts of 5 dpf wild type and *snap29*^{K164*} mutant larvae. Quantification of LC3II band intensity normalized with Vinculin (an aspecific band is marked by an asterisk), relative to wild type is reported. The LC3II protein level is increased by 1.31 times, compared to wild type. Uncropped blots are shown in Fig. S7A. (B) Detection of the autophagy adaptor marker p62 protein by Western blot in extracts of 4 dpf wild type and *snap29*^{K164*} mutant larvae. Quantification of p62 band intensity normalized with Vinculin (which shows an aspecific bands marked by an asterisk) relative to wild type is reported. p62 protein level is increase by 1.44 times compared to wild type. Quantifications were performed using Image Lab software. Uncropped blots are shown in Fig. S7B. (C) Paraffin transversal sections of 4 dpf wild type and *snap29*^{K164*} mutant forebrain stained with p62. High magnifications show punctate accumulation of p62 dots in *snap29*^{K164*} mutant compared to wild type. FB: forebrain, OL: optic lobe. (D) Electron microscopy cross-sections of 7 dpf larval skin. *snap29*^{K164*} mutants show presence of multilamellar organelles (MLOs) within extracellular cavities (red asterisk). MLO: multilamellar organelle, Mit: mitochondria, Nu: nucleus, BM: basement membrane.

Figure S6. Skin alterations in *snap29* mutant larvae

(A) Electron microscopy cross-sections of the epidermis of 7 dpf larvae. Pseudo-coloring in yellow identifies peridermal cells, in magenta basal layer cells and in orange basement membrane, respectively. *snap29*^{K164*} mutants show a thinner peridermal layer composed of elongated cells, compared to wild type, as well as a disrupted scale precursor pattern (red asterisks). SP: scale precursors, GA: Golgi apparatus, BM: basement membrane, Nu: nucleus. (A') Quantification of skin thickness measured in 7 dpf wild type and in *snap29*^{K164*} mutant larvae. The bars show mean and standard deviation. *P*-values obtained by unpaired t test. $^{**}P \leq 0.01$, n=4-12 sections. (A'') Quantification of scale precursors number per 10 µm of skin lenght measured in 7 dpf wild type and *snap29*^{K164*} mutant larvae. The bars show mean and standard deviation. *P*-values were obtained by unpaired t test with Welch's correction. $^{**}P \leq 0.01$, n=4-12 sections. (B) Detection of zebrafish Keratin protein by Western blot in protein extracts of 7 dpf wild type and *snap29*^{K164*} mutant larvae. The antibody recognizes a band at 58 kDa, which in *snap29*^{K164*} mutant is reduced by 61%. Quantification was performed using Image Lab software. Vinculin was used as loading control. The asterisk marks an aspecific band. Uncropped blots are shown in Fig. S7C. (C) High magnifications electron micrograph of epidermal cell junctions. *snap29*^{K164*} mutants shows malformed adherens junctions compared to wild type (white arrows) and presence of intercellular spaces (red asterisk), which are never found in wild type animals.

Figure S7. Proliferation in *snap29* mutant animals

(A) Comparable maximum projections of wild type and *snap29*^{K164*} mutant heads at 19 hpf, and 7 dpf stained with anti-pH3 to reveal presence of proliferating cells. White dashed-lines were drawn to highlight head morphology. (B) Quantification of the number of apoptotic cells per 10^5 μm^2, measured in wild type and *snap29*^{K164*} mutants at 19 hpf and 7 dpf, respectively. 19 hpf and 7 dpf *snap29*^{K164*} mutants show a significant increase of proliferating cells, when compared to wild type. The bars in the graph show means and standard deviations. Unpaired t-test with Welch's correction indicates that differences are not significant. n=2-6.

Figure S8. Complete blots.

(A) Uncropped blot relative to Fig. S5A. (B) Uncropped blot relative to Fig. S5B. HeLa cell extract was used as control to verify the correct molecular weight of p62 band. (C) Uncropped blot relative to Fig. S6B. Cropped areas are highlighted by red boxes.

List of supplementary movies

Movie S1: Touch evoked response of a representative wild type larva relative to Fig. 4C.

Movie S2: Touch evoked response of a representative *snap29*^{K164*} mutant relative to Fig. 4C.

Movie S3: Twitching per minute of a population of 26 hpf uninjected embryos relative to Fig. 5B.

Movie S4: Twitching per minute of a population of 26 hpf *snap29* morphants relative to Fig. 5B

Movie S5: Twitching per minute of a population of 26 hpf embryos injected with *GFP-snap29* mRNA relative to Fig. 5B

Movie S6: Twitching per minute of a population of 26 hpf *snap29* morphants injected with *GFP-snap29* mRNA relative to Fig. 5B

Movie S7: Twitching per minute of a population of 26 hpf *snap25* morphants relative to Fig. 5C

Movie S8: Twitching per minute of a population of 26 hpf *snap29* morphants relative to Fig. 5C.

Movie S9: Twitching per minute of a population of 26 hpf uninjected embryos relative to Fig. 5.

Movie S10: Twitching per minute of a population of 26 hpf *bdnf* morphants relative to Fig. 5C.
